# Precision Medicine Is Changing the Roles of Healthcare Professionals, Scientists, and Research Staff: Learnings from a Childhood Cancer Precision Medicine Trial

**DOI:** 10.3390/jpm13071033

**Published:** 2023-06-23

**Authors:** Rebecca Daly, Kate Hetherington, Emily Hazell, Bethany R. Wadling, Vanessa Tyrrell, Katherine M. Tucker, Glenn M. Marshall, David S. Ziegler, Loretta M. S. Lau, Toby N. Trahair, Tracey A. O’Brien, Kiri Collins, Andrew J. Gifford, Michelle Haber, Mark Pinese, David Malkin, Mark J. Cowley, Jonathan Karpelowsky, Donna Drew, Chris Jacobs, Claire E. Wakefield

**Affiliations:** 1 Discipline of Pediatrics and Child Health, School of Clinical Medicine, UNSW Medicine & Health, UNSW Sydney, Sydney, NSW 2052, Australia; 2 Behavioural Sciences Unit, Kids Cancer Centre, Sydney Children’s Hospital, Randwick, NSW 2031, Australia; 3 Graduate School of Health, University of Technology Sydney, Sydney, NSW 2007, Australia; 4 Children’s Cancer Institute, UNSW Sydney, Sydney, NSW 2052, Australia; 5 Hereditary Cancer Centre, Department of Medical Oncology, Prince of Wales Hospital, Randwick, NSW 2031, Australia; 6 Prince of Wales Clinical School, UNSW Sydney, Randwick, NSW 2031, Australia; 7 Kids Cancer Centre, Sydney Children’s Hospital, Randwick, NSW 2031, Australia; 8 Anatomical Pathology, NSW Health Pathology, Prince of Wales Hospital, Randwick, NSW 2031, Australia; 9 Division of Haematology/Oncology, The Hospital for Sick Children, Department of Paediatrics, University of Toronto, Toronto, ON M5G 1X8, Canada; 10 Kinghorn Centre for Clinical Genomics, Garvan Institute, Darlinghurst, NSW 2010, Australia; 11 Department of Paediatric Surgery, Children’s Hospital at Westmead, Westmead, NSW 2145, Australia; 12 Children’s Cancer Research Unit, Kids Research Institute, Children’s Hospital at Westmead, Westmead, NSW 2145, Australia; 13 Division of Child and Adolescent Health, University of Sydney, Sydney, NSW 2145, Australia

**Keywords:** precision medicine, pediatric, oncology, workforce, genomic sequencing

## Abstract

Precision medicine programs aim to utilize novel technologies to identify personalized treatments for children with cancer. Delivering these programs requires interdisciplinary efforts, yet the many groups involved are understudied. This study explored the experiences of a broad range of professionals delivering Australia’s first precision medicine trial for children with poor-prognosis cancer: the PRecISion Medicine for Children with Cancer (PRISM) national clinical trial of the Zero Childhood Cancer Program. We conducted semi-structured interviews with 85 PRISM professionals from eight professional groups, including oncologists, surgeons, clinical research associates, scientists, genetic professionals, pathologists, animal care technicians, and nurses. We analyzed interviews thematically. Professionals shared that precision medicine can add complexity to their role and result in less certain outcomes for families. Although many participants described experiencing a greater emotional impact from their work, most expressed very positive views about the impact of precision medicine on their profession and its future potential. Most reported navigating precision medicine without formal training. Each group described unique challenges involved in adapting to precision medicine in their profession. Addressing training gaps and meeting the specific needs of many professional groups involved in precision medicine will be essential to ensure the successful implementation of standard care.

## 1. Introduction

Precision medicine is evolving childhood cancer treatment, with multiple large-scale trials enrolling patients worldwide [1]. Precision medicine moves away from a “one-size-fits-all” approach, instead examining the molecular characteristics of each patient’s tumor [2,3]. Precision medicine trials aim to identify more effective and less toxic therapies by utilizing techniques such as whole-exome and whole-genome sequencing. Some trials also use patient-derived xenograft (PDX) models and in vitro drug testing to increase the chances of identifying novel therapeutics [4,5,6,7]. Precision medicine is increasingly becoming part of routine clinical care for pediatric cancers [1,8]. Healthcare professionals have largely positive attitudes towards precision medicine in oncology [9], but it is not yet clear whether the workforce is adequately prepared to meet future demands [10].

Precision medicine trials provide a wealth of information for clinical teams to integrate into patient care. However, healthcare professionals can feel underequipped to interpret these data [11]. Oncologists have reported difficulty understanding and communicating genomic information and managing families’ emotional responses and expectations [12,13,14]. Surgeons have described a lack of confidence in discussing genetic testing and results with patients with pathogenic variants in BRCA1/BRCA2 [15,16], and nurses have stated having a limited knowledge of genomics, despite acknowledging its increasing importance [17]. Precision medicine has resulted in a growing demand for and reliance on genetic counsellors and geneticists (hereby referred to as genetic professionals) [18,19,20], who, despite their specialized training, are reporting increased complexity and diversity in the patient phenotypes and data they encounter [20].

Precision medicine also involves professionals working “behind the scenes” in non-patient-facing roles, including pathologists, scientists, animal care technicians, and clinical research associates (CRAs). Their experiences are currently understudied. Animal care technicians are responsible for the health and welfare of PDX mouse models, which are contributing to the advancement of the knowledge and improvement of therapies [21,22]. CRAs are involved in all aspects of running clinical trials and can offer unique perspectives on physician-, patient-, and system-related factors that may influence trial implementation [23]. For pathologists, precision medicine technologies have driven a diagnostic shift from morphology to molecular pathology [24,25,26,27], yet little is known of their perspectives.

To ensure the effective implementation of precision medicine, it is important to consider a wide range of stakeholders’ views [28]. We previously explored the early experiences of oncologists and scientists in the first year of delivering a precision medicine trial for children with high-risk cancer [12] but did not examine the experiences of other important professional groups. To address this gap and build upon our previous work, we continued to track the experiences of oncologists and scientists as the trial progressed and expanded our recruitment to capture the perspectives of understudied groups such as surgeons, nurses, animal care technicians, and CRAs. Recognizing these stakeholders’ viewpoints and experiences is critical to support the development of a broad workforce managing the demands of pediatric precision medicine [12].

## 2. Materials and Methods

### 2.1. PRISM and PRISM-Impact

The PRecISion Medicine for Children with Cancer (PRISM) study is a national multicenter precision medicine clinical trial for children and adolescents (≤21 years) with poor-prognosis cancers in Australia embedded in the Zero Childhood Cancer Program [28,29] (Australian and New Zealand Clinical Trials Registry (NCT03336931)). PRISM-Impact is a mixed-methods longitudinal psychosocial study that runs alongside the PRISM trial. PRISM and PRISM-Impact were conducted in accordance with the Declaration of Helsinki and received institutional board approval (ethical and governance approval number: HREC/17/HNE/29).

### 2.2. Participants

**Figure 1:**
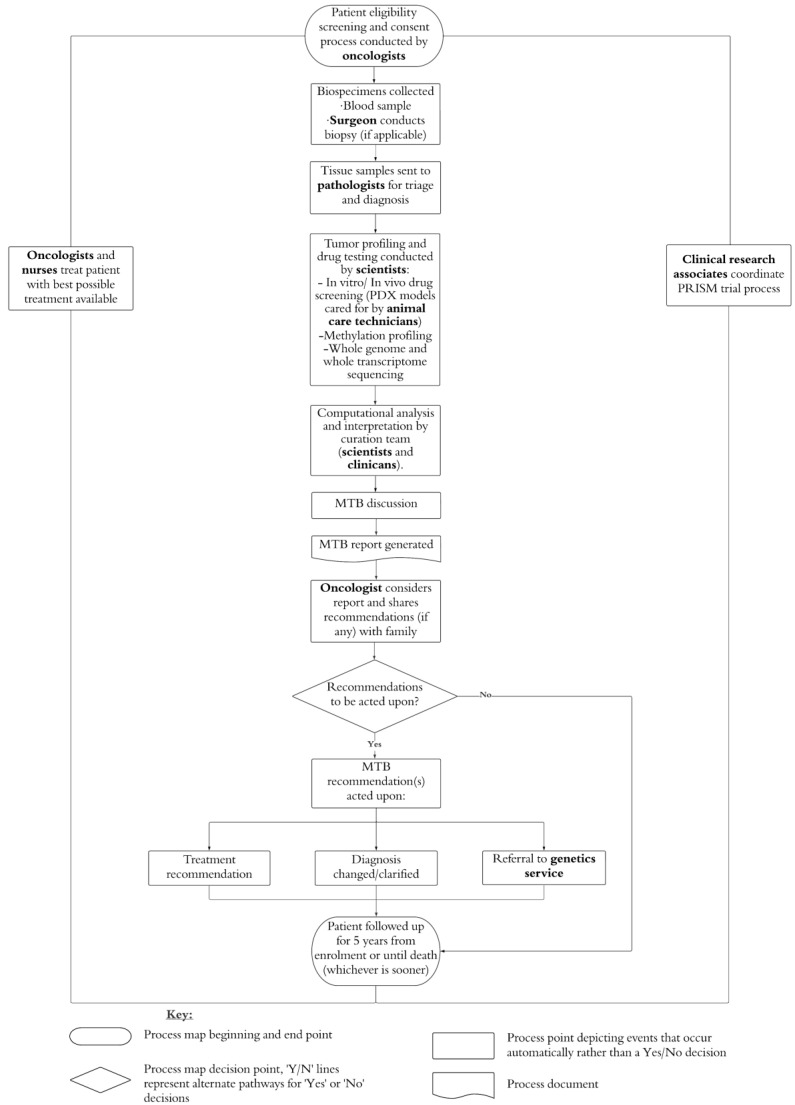
PRISM trial processes with professional roles highlighted. Note: PDX = Patient-derived xenograft; MTB = multidisciplinary tumor board.

We invited healthcare professionals (oncologists, surgeons, pathologists, oncology nurses, and genetic professionals), scientists, and research staff (animal care technicians and CRAs) to participate in a semi-structured interview. Participants were eligible if their role involved working on PRISM. We used purposive sampling to gather breadth of experience from all groups. Figure 1 summarizes the PRISM trial and the roles of the patient-facing and non-patient-facing professionals we recruited.

### 2.3. Procedure

Potential participant lists were provided by the PRISM coordinating team. Participants were invited via a personalized email from the study chair (DZ), the surgical lead in PRISM (JK), or a psychosocial researcher (RD, KH) during the second and third year of the trial. Participants opted in or out via email reply. We sent follow-up emails if participants did not respond within two weeks of receiving the initial invitation. Participants who could not be reached after three failed follow-up attempts were deemed unreachable. Participants could choose to complete their interview in person (if based in Sydney, Australia) or by telephone. Three psychosocial researchers with post-graduate degrees (RD, JDH, and NH) conducted in-depth semi-structured interviews, which lasted 25 min on average (range: 11.34–51.46 min). Interviews were audio-recorded and transcribed verbatim with identifiable information (i.e., participant names and hospital site), which was removed prior to analysis.

### 2.4. Interview

The semi-structured interview schedule was developed with input from a multidisciplinary team comprising oncology healthcare professionals, psychosocial researchers, and a prominent member of each of the eight professional groups interviewed. Topics included experiences with PRISM, hopes and expectations of precision medicine, experiences of a multidisciplinary tumor board (MTB), training and support needs, confidence, and knowledge. Interview schedules were adapted for each professional group to ensure that only questions relevant to the participants’ role were asked (Supplementary Table S1). All participants provided informed consent.

### 2.5. Data Analysis

We analyzed participants’ demographic data with the Statistical Package for the Social Sciences (SPSS version; IBM, Armonk, NY, USA). We analyzed qualitative data using an inductive thematic approach [30]. For each sub-group, one researcher (RD) became familiarized with the data and developed an initial coding framework [30]. The initial coding framework was discussed, revised, and further developed via discussion with two researchers (CW and KH). Interviews were then coded line by line, reviewed, and revised, and any disagreements were discussed until consensus was reached.

## 3. Results

### 3.1. Participants

We invited 185 professionals, of whom 101 opted in (54.5% response rate) with 85 participants completing an interview (84.1% participation rate), as shown in Table 1. Participants were 50% female, 43.1 (±11) years old on average (range: 24–75),and working across eight metropolitan hospitals and one institute. Ten (11.7%) participants were investigators on the PRISM study. Twenty-five participants (29.4%) were based at Sydney Children’s Hospital. Twenty-six oncologists, thirteen surgeons, twelve CRAs, ten scientists, ten genetic professionals, five pathologists, five animal care technicians, and four nurses were interviewed between September 2018 and November 2020 (Table 2). At the conclusion of recruitment for this study (November 2020), the PRISM trial had enrolled 401 patients (Supplementary Table S2).

### 3.2. Themes

**Figure 2:**
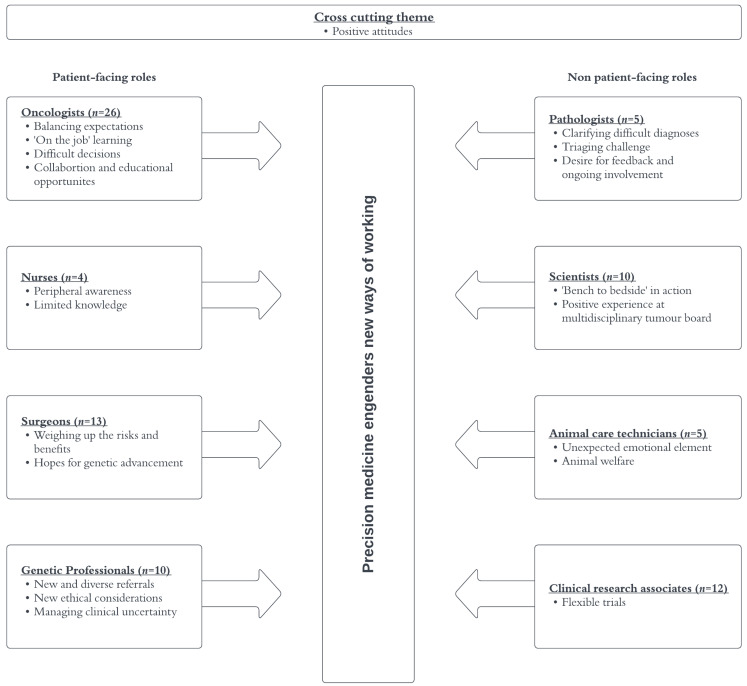
Summary of themes from interviews with PRISM professionals.

Figure 2 summarizes the themes that emerged from the interviews. We describe each theme organized by professional groups below and illustrate our findings with representative quotes in Supplementary Table S3.

#### 3.2.1. Cross-Cutting Theme

Positive attitudes: Professionals across all groups held positive attitudes towards precision medicine and working on PRISM. Many participants valued being involved in the trial citing the potential outcomes for patients, learning opportunities, and the reassurance provided for families in delivering possible treatments for their child.

#### 3.2.2. Oncologists

Balancing expectations: Oncologists emphasized the importance of ensuring that parents were properly informed at the time of trial consent, as some families had “challenging to manage” high expectations of precision medicine.

“On the job” learning: Most oncologists shared that they did not have formal genetics training. Some perceived that they were “deficient” in their knowledge of, or had “no idea” about, precision medicine when they first became involved in the trial; however, they believed that this improved while working on the trial or initiating independent learning.

Difficult decisions: Oncologists described difficulties regarding whether to action recommendations emerging from the MTB. They felt a new responsibility associated with assessing the weight of the available evidence and often faced barriers to implementation, such as a lack of evidence and poor drug access: *“Sometimes there are no answers because while you might find a target, there aren’t any drugs yet. It’s hard to comprehend some of that.”* (Oncologist 008).

Collaboration and educational opportunities: Oncologists described precision medicine as promoting strong collaborations between disciplines, particularly via the MTB meetings. Many oncologists shared that having input from other disciplines helped to improve their knowledge and understanding of complex findings and informed clinical decision-making. Additionally, precision medicine resulted in oncologists collaborating closely with genetic professionals to convey the meaning of precision medicine to families, particularly regarding germline findings.

#### 3.2.3. Nurses

Peripheral awareness: Most nurses shared that precision medicine had little or no impact on their patient care or day-to-day role. They were peripherally aware of the trial but described it as happening in the “background”: *“There is definitely a disconnect between nurses being aware of what’s happening and what the study means”* (Nurse 082). One nurse who was actively involved in patient enrolment to the trial described families as having high expectations of precision medicine and depicted interactions with these families as requiring extra time to align their expectations with possible outcomes.

Limited knowledge: Nurses shared that, while they usually knew which of their patients were participating in drug trials in the ward, they were less aware of those families involved in PRISM. Nurses described learning that a patient was participating in precision medicine when a family asked them about receiving results or about the trial process. When this occurred, nurses found it challenging to answer questions and would refer families to their oncologist.

#### 3.2.4. Surgeons

Weighing risks and benefits: Surgeons described weighing the risks and benefits of obtaining large enough samples to enable participation in the trial: *“I for one would not subject the patient to an operation [for PRISM] that had much greater risk of morbidity if you know a core biopsy for instance could do it more safely.”* (Surgeon 089) They described feelings of increased pressure or “a push” to obtain sufficient tissue during biopsy procedures to facilitate the testing. Some felt this was reinforced by experiences of negative feedback or disappointment from the wider precision medicine team when a sample obtained was not sufficient.

Hopes for genetic advancement: Surgeons felt precision medicine had potential benefits for families, including for identifying additional treatment options and empowering families with the provision of more information about their child’s cancer. Some also expressed hope that genetic advances would result in treatments with less morbidity in the future.

#### 3.2.5. Genetic Professionals

New and diverse referrals: Genetic professionals acknowledged an increased demand for professionals with formal genetic training. They shared that genetic referrals were previously based on family history or clinical features and involved limited gene panels, whereas PRISM involved a new model of genetic care offering germline whole genome sequencing to all enrolled patients.

New ethical considerations: Some genetic professionals felt that, despite parents giving informed consent, some were not fully prepared or had not considered the potential long-term or family-wide implications of receiving germline findings from PRISM. Genetic professionals shared that working in precision medicine had resulted in some complex ethical dilemmas, for example, when an actionable germline finding was identified, when the parents had not consented to receiving the result, or when results became available after the child died.

Managing clinical uncertainty: Genetic professionals recognized that the rapid development of precision medicine research meant that they were sometimes required to interpret complex findings. Such findings involved clinical uncertainties and often no standardized surveillance protocols, which they found difficult to explain to families.

#### 3.2.6. Pathologists

Clarifying difficult diagnoses: Pathologists shared that precision medicine testing had been beneficial, allowing them to clarify difficult cases and refine existing diagnoses in childhood cancer, which would have previously not been possible. Pathologists acknowledged that their profession is gradually moving into the “molecular world,” where diseases are classified genomically as well as histopathologically, and that precision medicine was *“going to become more and more standard part of diagnosis work”* (Pathologist 068).

Triaging challenges: Pathologists shared that ensuring sufficient samples were available for routine testing and trial participation was challenging. Pediatric biopsy samples are typically small, and some pathologists reported that the sample requirements for the trial were sometimes not achievable. On occasion, there were insufficient samples for PRISM testing, which pathologists suspected was disappointing for families and the treatment team. Pathologists reported that they did not routinely receive information on whether samples sent for testing were sufficient, with many expressing a desire for such feedback to improve future sample provision.

Desire for feedback and ongoing involvement: Some pathologists also expressed a desire to receive feedback about the clinical outcomes of individual patients and shared an eagerness to learn more from the trial results.

#### 3.2.7. Scientists

“Bench to bedside” in action: Scientists described being involved in PRISM as an exciting opportunity to contribute to translational science. Scientists also described benefiting from being exposed to clinical decision-making based on their work and from seeing their work having direct patient outcomes. However, this direct link to patients also resulted in additional time pressures to produce results, which was amplified by knowing families were waiting.

Positive experience at the MTB: Scientists found participating in the MTB to be a positive educational experience and valued the opportunity to collaborate with patient-facing colleagues and build new professional networks.

#### 3.2.8. Animal Care Technicians

Unexpected emotional elements: Many animal care technicians described a new and unexpected emotional element to their work: if an animal linked to a patient died, some animal care technicians disclosed feeling disheartened or upset. Some shared that working on the trial was more difficult than other projects as those projects did not have direct implications for an individual patient. Knowing that animals were directly linked to a patient created a heightened sense of significance, which increased feelings of urgency, stress, and responsibility.

Animal welfare: Although acknowledging the clinical importance of their work for patients, animal care technicians were clear that their top priority was always animal welfare and described advocating for animals’ needs: *“If an animal is suffering, I send an e-mail to the researchers saying that it must be culled…. Even though that animal may be linked to a patient, it may be critical, in this case the animal comes first”* (Animal care technician 080).

#### 3.2.9. Clinical Research Associates (CRAs)

Flexible trials: CRAs described the work on PRISM as rewarding, and they shared their excitement about the potential clinical benefits. However, they also described some unique challenges delivering precision medicine trials compared with traditional trials. CRAs described traditional trials as having clearer data entry points, on-site monitoring, and mandated follow-up requirements, whereas PRISM was viewed as requiring more flexibility. Due to the heterogenous nature of the cohort in terms of diagnosis, disease stage, and age, CRAs also shared that organizing the recommended three monthly follows-ups was more complex than organizing follow-ups in traditional pharmaceutical trials.

## 4. Discussion

This qualitative study is the first to assess the experiences of a diverse cohort of professionals involved in delivering a precision medicine trial. We offer the unique insights of animal care technicians, pathologists, surgeons, CRAs, and nurses whose perspectives of pediatric precision medicine have not been captured previously. We explored how precision medicine is changing professional roles and challenges encountered. Echoing previous research [9], most professionals described positive experiences with the PRISM study and the potential applications of precision medicine in childhood cancer. Participants across groups valued the availability of precision medicine testing in Australia and providing reassurance to families that the study was exhausting every avenue to treat patients with a poor prognosis. Non-patient-facing professionals described an increased sense that their work was having a real-world impact, which they found rewarding. On an individual level, we found that professionals across different groups experienced additional pressures and an emotional burden associated with working on the trial. Participants from most groups described gaps in their knowledge regarding precision medicine, and some desired stronger collaboration and communication between disciplines.

Similarly to our previous study [12] we found that precision medicine trials are resulting in a new way of working for the many professionals involved. Scientists, animal care technicians, and CRAs are drawn closer to the clinical interface resulting in not only an additional pressure to produce results and an emotional burden but also a rewarding sense that their work is having a real-world impact. The incorporation of advances in precision medicine is revolutionizing the disciplines of pathology and genetics and changing professionals’ traditional approaches to cancer care. In contrast, nurse participants had little knowledge of PRISM and shared that precision medicine had little or no impact on their approach to patient care. Given nurses’ frequent, direct contact with patients and families, they have the potential to act as educators [31], an opportunity which appears to be currently underutilized. Surgeons and pathologists can experience pressure to maximize biopsy samples for testing, despite obtaining sufficient samples for functional testing being a recognized roadblock in pediatric trials [32]. In line with other studies, professionals involved in the PRISM MTB found it a positive experience, which offered educational opportunities [33], collaboration, and an ability for laboratory-based staff to see the potential clinical value in their work.

Our findings also revealed that precision medicine is adding complexity to decision-making, particularly for oncologists and genetic professionals as they struggle with the growing gap between clinical knowledge and genetic potential [34]. This was particularly salient when professionals discussed communicating with families about their expectations, outcomes, and results. Future research should further explore the clinical decision-making process involved in implementing novel treatments recommended by precision medicine and reporting and communicating identified genetic risks.

A key insight that emerged during this analysis was that most professionals are navigating precision medicine without formal training, despite an appetite to learn more. This suggests varied knowledge and confidence across our participants and their respective disciplines, reinforcing the findings of our earlier study [12]. This signals a growing need for precision medicine education for professionals, with discipline-specific resources and opportunities for ongoing learning in this rapidly evolving field.

Participants were keen to learn more about precision medicine and the trial outcomes, highlighting the importance of the interdisciplinary dissemination of findings. Professionals in non-patient-facing roles who did not regularly attend the MTB expressed a desire to receive feedback about clinical outcomes. Our findings recommend that precision medicine programs promote stronger interdisciplinary collaboration and communication.

A strength of our study was the inclusion of a diverse sample of professional stakeholders. Despite this, the response rate of some cohorts was low (Table 1). This could reflect a lack of knowledge regarding the trial in some professional groups, meaning that some potential participants may have felt unable to contribute to our study. Our pool of potential participants was limited as PRISM is the first precision medicine trial for children with poor-prognosis cancer in Australia. Some participants were study investigators for the trial, and our sample included a larger proportion of professionals from Sydney Children’s Hospital. This may have influenced their perspectives on precision medicine and limited the generalizability of our findings. Future research should examine the experiences of professionals in international pediatric precision medicine studies and trials with patients with good prognosis cancers as precision medicine expands further into the standard of care for childhood cancer. The application of precision medicine is expected to grow exponentially with time and continued investment [2]. Our study provides new insights into the impact of precision medicine on a diverse population of professionals, including groups not studied before. Our findings highlight the need for role-specific training and educational resources. Many participants in our study were trained before the emergence of genomic medicine. Updating educational curriculums to include genomics will help to better prepare professionals for precision medicine [35]. As genetics is a rapidly evolving area, continuous educational opportunities are essential to ensure that current and future workforces keep up to date with developments [35]. Strategies that may increase knowledge of precision medicine across disciplines include freely accessible online education presented in multiple formats, collaborative interdisciplinary forums, industry forums, and the provision of information and educational materials via multiple channels [36]. Participants who attended the PRISM MTB found it to be a positive and educational experience. Exploring ways to maximize the MTB’s educational potential (e.g., capturing clinical learnings) would be beneficial. Our study highlighted the added emotional element precision medicine can bring to some roles such as scientists and animal care technicians. Given animal care technicians are already known to be at risk of experiencing compassion fatigue [37], it is important to recognize the potential emotional impact that working on a precision medicine trial may have and to provide appropriate support. The encouragement of a culture of openness regarding emotional labor, workplace support, and initiatives such as resilience training could help protect and improve staff wellbeing in this role [38]. Our findings also support the promotion of multidisciplinary models of care and inform the development of ethically defensible plans to help guide the management and return of genetic results to families. Meeting the specific training needs and challenges revealed in this study will be essential to ensure successful implementation in the future.

## 5. Conclusions

Healthcare professionals, scientists, and research staff involved in precision medicine programs are adjusting to a new approach to cancer care while managing role-specific challenges.

## Figures and Tables

**Table 1:** Participation and response rates of PRISM-Impact.

Profession	Invited (*n*)	Opted in (*n*)	Participated (*n*)	Response Rate (*%*)	Participation Rate (*%*)
Oncologist	57	30	26	52.6	86.6
Surgeon	21	17	13	80.9	76.4
Clinical Research Associate	24	13	12	54.1	92.3
Genetic Professionals	15	10	10	66.6	100
Scientist	24	11	10	45.0	90.9
Pathologist	22	7	5	31.8	71.4
Nurse	15	6	4	40.0	66.6
Animal Care Technician	9	7	5	77.7	71.4
Total	187	101	85	-	-

Note: Response rate = number of individuals who opted in/number of individuals invited to participate ×100. Participation rate = number of individuals who participated/number of individuals who opted in × 100.

**Table 2:** Participant demographics.

Characteristic	Participants (*n* = 85)
Profession, *n*	
Oncologist	26
Surgeon	13
Clinical Research Associate	12
Genetics Professional	10
Scientist	10
Pathologist	5
Nurse	4
Animal Care Technician	5
Site, *n* (%)	
Sydney Children’s Hospital	25 (29.4)
Children’s Cancer Institute	15 (17.6)
Royal Children’s Hospital, Melbourne	9 (10.6)
Perth Children’s Hospital	9 (10.6)
Queensland Children’s Hospital	8 (9.4)
The Children’s Hospital, Westmead	8 (9.4)
John Hunter Children’s Hospital, Newcastle	5 (5.9)
Women’s and Children’s Hospital, Adelaide	4 (4.7)
Monash Children’s Hospital, Melbourne	2 (2.4)
Age (years), Mean (*SD*), Range	43.1 (11), 24–75
Gender, *n* (%)	
Female	50 (58.8)
No. years working in pediatric oncology by profession, mean (*SD*), range ^1^	
Oncologist	15.61 (10.9), 6–50
Nurse	17.25 (12), 6–30
Genetics Professional	12.93 (14.2), 1–40
Clinical Research Associate	4.20 (4.1) 0–14
No. years of practice by profession, mean (*SD*), range ^1^	
Pathologist	17.13 (9.5), 9–30
Scientist	8.52 (10), 1–30
Animal Care Technician	8.60 (9.5), 1–25
Percentage of their time dedicated to research by profession, mean (*SD*), range ^1^	
Oncologist	35.4 (27.7), 5–100
Nurse	37.5 (12.5), 20–50
Surgeon	8.71 (7.6), 2–30
Genetics Professional	22.75 (23.9), 2–60
Pathologist	8.4 (7), 2–20
Scientist	100 (100), 100–100
Animal Care Technician	47.5 (33), 10–90

^1^ Item not administered to all groups. *SD* = standard deviation; *n* = number.

## Data Availability

The data presented in this study are available on request from the corresponding author. The data are not publicly available due to privacy/ethical reasons.

## Supplementary Materials

The following supporting information can be downloaded at https://www.mdpi.com/article/10.3390/jpm13071033/s1. Table S1: Healthcare professionals’ semi-structured interview guide; Table S2:PRISM hospital site recruitment (November 2020); Table S3: Themes with representative quotes.
