# Supplementary material for: Precision Medicine Is Changing the Roles of Healthcare Professionals, Scientists, and Research Staff: Learnings from a Childhood Cancer Precision Medicine Trial
Source: J Pers Med. 2023 Jun 23;13(7):1033. doi: 10.3390/jpm13071033 (PMC10381580; doi:10.3390/jpm13071033)
Supplement: Supplementary file 1 [file jpm-13-01033-s001.zip › Supplementary Table S1.pdf]

Supplementary Table S1: Healthcare professionals' semi-structured interview guide

Questions and prompts	Clinicians	Scientists	CRA	ACT	Pathologists	Nurses	Genetics Professionals
Demographics							
- Date of birth<br>- Gender<br>- Professional title<br>- What percentage of your time is dedicated toward research?<br>- What percentage of your time is dedicated towards PRISM?	x	x	x	x	x	x	x
Experiences with PRISM							
- What was your role with patients and how did you interact with them regarding the PRISM study?	x		x			x	x
- What is your experience of being part of the PRISM study so far?	x	x	x	x	x	x	x
- What positive experiences (of interacting with families) regarding PRISM/have you had working with PRISM (if any)?	x	x	x	x	x	x	x
- Have you had any negative experiences?	x	x	x	x	x	x	x
- What do you think are the key advantages of PRISM for families?	x	x	x	x	x	x	x
- What do you think are the key disadvantages of PRISM for families?	x	x	x	x	x	x	x
- Have you experienced any barriers to involving families in PRISM?	x						x
- What are the most difficult parts and challenges of PRISM, from your perspective?	x	x			x	x	
- Does your work on the precision medicine trial differ to other trials?		x	x	x	x		
- Conducting new research testing such as that offered by PRISM requires a change in usual practice, for example taking a new biopsy at relapse or taking a larger biopsy. What is your impression of parent's willingness to do this in a safe manner, particularly when there is no guarantee that it will benefit their child?<br>- Do you think this is appropriate?	x				x	x	
Treatment recommendations							
- Have any of your patients [cases] received treatment recommendations from PRISM?	x				x	x	
- Have any of your patients received germline genetic test results? Were there any benefits or difficulties you can think of? Were the results what you expected?	x					x	

- Have you personally communicated germline results to families? What was your experience? Prompt: positive and negative. If yes, did you feel comfortable sharing germline results?	x					x	
- Did you notice differences in sharing results (working with PRISM patients) from PRISM compared with other medical results? Do you think it is different to any other situation in clinical practice?	x					x	
Training and support							
- How much genetic specific training have you had?	x	x			x	x	
- Do you feel your confidence has changed in explaining somatic/germline results from working on the study and your knowledge in general?	x	x			x	x	
Multidisciplinary tumour board							
- Are you part of the MTB?<br>- If yes, what has your experience been so far?	x	x	x		x		x
Ethics of trial/precision medicine technology							
- What is your opinion about sharing raw genomic data with families?<br>- Would you hand it out to families or would you rather not?	x				x		x
- How much time does it usually take to analyse the PRISM samples? (Average length to analyse all samples for each patient)<br>- Probe: Do you think this is an appropriate amount of time<br>- If answer is too long: how could process, be accelerated?		x	x	x			
Profession specific questions							
- How do you balance the welfare of the animal with the potential patient outcomes?				x			
- How do you balance the welfare of the animal with the potential patient outcomes?				x			
- What are your views on how the germline information was presented at the MTB?							x
- What is your opinion about sharing information about recessive conditions back to families which may only be useful for family planning but not related to the child's disease?							x
- What do you think needs improved in the future for trials such as PRISM?			x				
- What do you think needs improved in the future for trials such as PRISM?			x				
- Can you please describe your role in PRISM including your day-to-day duties?			x				
- Do you routinely send all the required biopsy material? If not, why?					x		
- Do you ever receive feedback on molecular findings from the PRISM cases you have reported?					x		

- Have you personally communicated germline results to families? What was your experience? Prompt: positive and negative. If yes, did you feel comfortable sharing germline results?	x						
- Were you involved in sharing the PRISM results with parents/patients?<br>- How confident did you feel about your understanding of the PRISM results?<br>- How comfortable did you feel explaining these results to parents/patients?<br>- If yes, what do you think could improve the way this information is shared with parents/patients (if any)?	x						
