# Supplementary material for: Precision Medicine Is Changing the Roles of Healthcare Professionals, Scientists, and Research Staff: Learnings from a Childhood Cancer Precision Medicine Trial
Source: J Pers Med. 2023 Jun 23;13(7):1033. doi: 10.3390/jpm13071033 (PMC10381580; doi:10.3390/jpm13071033)
Supplement: Supplementary file 1 [file jpm-13-01033-s001.zip › Supplementary Table S2.pdf]

**Supplementary Table S2. PRISM hospital site recruitment (November 2020)**

| **Site** | **Patients, n (%)** |
|---|---|
| Royal Children’s Hospital, Melbourne | 76 (18.7) |
| Sydney Children’s Hospital | 75 (18.7) |
| The Children’s Hospital, Westmead | 75 (18.7) |
| Queensland Children's Hospital | 57 (14.2) |
| Perth Children's Hospital | 43 (10.7) |
| Monash Children's Hospital, Melbourne | 34 (8.5) |
| John Hunter Children's Hospital, Newcastle | 25 (6.2) |
| Women’s and Children’s Hospital, Adelaide | 16 (4) |
| Total | 401 |
