# Supplementary material for: Precision Medicine Is Changing the Roles of Healthcare Professionals, Scientists, and Research Staff: Learnings from a Childhood Cancer Precision Medicine Trial
Source: J Pers Med. 2023 Jun 23;13(7):1033. doi: 10.3390/jpm13071033 (PMC10381580; doi:10.3390/jpm13071033)
Supplement: Supplementary file 1 [file jpm-13-01033-s001.zip › Supplementary Table S3.pdf]

Supplementary Table S33. Themes with representative quotes

Profession	Theme	Quotes	ID
**Cross cutting theme**	Positive attitudes	*'Families can be confident that they've had the best possible care for their children, by going through PRISM. To be confident that no stone has been left unturned, with the most comprehensive testing program available almost anywhere in the world' [Scientist]*	043
		'For the families PRISM gives them the peace of mind that they've done everything they can, to try and do the best thing by their child' [Pathologist]	053
		'I think that it is really good for us to be able to offer this type of testing here in Australia, and not have to say to families "Oh, we can't do this here". I think the testing and technology is sort of pretty world-leading, so I think that Australian paediatric oncologists have benefited from that". [Oncologist]	055
		'We are picking up a lot more actionable findings than I had anticipated and definitely seeing more results in terms of actual drug efficacy in patients than I had anticipated' [Oncologist]	016
		'I think the most important thing was to feel as though, no stone had been left unturned, that [families] had done everything that they possibly could' [CRA]	070
**Oncologists**	Balancing expectations	*'Ultimately trying not to undersell or oversell the possibilities with it. I think there's a big potential for therapeutic misconception at every level of the study. I've been keen to try and make sure that people understand it's about generating information in research that's probably more likely to be useful in the future but may potentially be useful for individuals and families as well.'*	059
	'On the job' learning	*'From a knowledge perspective, I realised how deficient I was at the beginning. We are always equipped with the basics, but PRISM is a whole new ball game. It is a completely, incredibly, high end, deep kind of investigation into all this and this just reflects how poor – how superficial the training programs are, and it was like people spoke 'Klingon' you know, around the table and you had no clue what they were speaking'*	060
	Difficult decisions	*'It's written down that you can try this drug, but in fact, the evidence for that drug is poor. The safety for those drugs is poor. The access to those drugs is poor. And you then have to decompress that to the families so that they understand that in no way am I ever giving your child this drug.'*	028
	Collaboration and educational opportunities	*'The more time I've spent being part of the MTB, the more I sort of understood the role and being able to participate. You know a bit better understanding of pharma, what's available, trials. So, I think we've, all of us have built up expertise and knowledge in that area'*	008

**Nurses**	Peripheral awareness	*'I think it's great don't get me wrong I think that there is definitely a disconnect between nurses being aware of what's happening and what the study means'*	082
	Limited knowledge	*'I just say look, I don't know enough about it, and it's something you need to speak to your consultant about. Then I'll e-mail the consultant or the team and go "met with this family today, wanting to know issues around PRISM or when will results be" and that would be what I'd do.'*	074
**Surgeons**	Weighing up risks and benefits	*'We offer core needle biopsies which would be standard of care here. But in some cases, many cases for PRISM, possibly we would need to possibly take more invasive biopsies depending on how big the risk for that is would depend on whether we go ahead'*	002
	Hopes for genetic advancement	*'I hope very much that the results of the study are realised and if you're successful in your endeavours my job becomes obsolete. I won't have to operate on anybody who has cancer because you'll be able to target the mutations and block it off with drugs.'*	084
**Genetics professionals**	New and diverse referrals	*'We've gone from somatic results barely coming up in our work as cancer genetics people to having more and more germline results that come from a somatic beginning'*	099
	Managing clinical uncertainty	*'For example, a child that had brain tumour, they have a gene associated with breast cancer and then how does the family fit all that information and how do they make sense of that?... I think partly we don't understand all of the tumours that might be involved in you know all of the – all of the problems that faulty genes can cause. So, you know, maybe in 12 years' time or something we might know more but I think at the moment we just said that it's likely to be contributory but unlikely to be the single cause of the child's cancer, but I guess for the wider family that was a bit of a bombshell'*	062
	New ethical considerations	*'There was one family that didn't consent to germline findings, but we did find the pathogenic mutation in that child and so now I don't know what to do with that, it's important for the families to know, but they've made it quite clear that they don't want to.'*	005
**Pathologists**	Clarifying difficult diagnoses	*'We've certainly had some cases that have been really complex and difficult in getting that additional molecular data or genetic information from PRISM, even if it's down the track. It has actually really helped quite a lot and there's one case in particular I can think of where it really has clarified something which we've really struggled with.'*	069
	Triaging challenge	*'We may only have limited material, so the challenge is to work out how to divide up that sample for all the necessary testing'*	053
	Desire for feedback and ongoing involvement	*'Very little [feedback] comes back to us, you know, I know it's a research project but, you know, something like 50-60% of the [hospital] patients if not more end up on research projects, it almost is actually standard of care so not getting so much in the way of feedback at all as to what results have been found, even if they're never acted upon, even if they never go into our databases, it's still very useful to have that sort of feedback purely from our own educational point of view if nothing else'*	075

**Scientists**	'Bench to bedside' in action	*'It's nice to actually be a bit more directly involved in some of the patient care rather than something that's decades away potentially from patient care and I think the…the sort of more direct involvement in providing information that goes towards this decision making'*	041
	Positive experience at MTB	*'You actually learn how the clinician is managing the patients, and you know the real time status of the patients, and you sort of – it's a really valuable experience for me to learn how they make those clinical decisions'*	031
**Animal-care technicians**	Unexpected emotional element	*'You do feel the pressure with every animal is so important that if something does go wrong then you do feel a bit, like, responsible and all that kind of stuff and this stuff does cost money, every animal in there is valuable and so if something happens then it, yeh it's hard, it's tough, it can be tough.'*	079
	Maintained focus on animal welfare	*'Our modus operandi for our jobs, our duty of care to those animals, we don't have any scopes on the patients. Obviously, the duty of welfare is for the animals because we don't want them to be in pain while they are in our care. We want them to live the best life that they can under the circumstances they are living. Now if we know it's a precision medicine study, we know it's important so we're going to make sure that you know that the animal's survival, that if anything's sick we've caught it as early as possible, so tissue is not wasted so that's an area that's really important for us.'*	077
**Clinical research associates**	Flexible trials	*"I think one positive aspect of PRISM is when you're sitting in the molecular tumour board, you can actually see results being communicated and a really good discussion between clinicians. From a study coordinator's perspective, I don't think you usually see that or have the opportunity to see that in trials. We oversee the data being entered by all the sites, we get to see almost firsthand where the treatment – where a recommended treatment has – data's been entered – ok they took it up and they're responding well and that's very rewarding."*	067
